# A receptor-binding domain-based nanoparticle vaccine elicits durable neutralizing antibody responses against SARS-CoV-2 and variants of concern

**DOI:** 10.1080/22221751.2022.2149353

**Published:** 2022-12-24

**Authors:** I-Jung Lee, Yu-Hua Lan, Ping-Yi Wu, Yan-Wei Wu, Yu-Hung Chen, Sheng-Che Tseng, Tzu-Jiun Kuo, Cheng-Pu Sun, Jia-Tsrong Jan, Hsiu-Hua Ma, Chun-Che Liao, Jian-Jong Liang, Hui-Ying Ko, Chih-Shin Chang, Wen-Chun Liu, Yi-An Ko, Yen-Hui Chen, Zong-Lin Sie, Szu-I Tsung, Yi-Ling Lin, I-Hsuan Wang, Mi-Hua Tao

**Affiliations:** a Graduate Institute of Microbiology, College of Medicine, National Taiwan University, Taipei, Taiwan; b Institute of Biomedical Sciences, Academia Sinica, Taipei, Taiwan; c School of Medicine, College of Medicine, National Cheng Kung University, Tainan, Taiwan; d Genomics Research Center, Academia Sinica, Taipei, Taiwan; e Biomedical Translation Research Center, Academia Sinica, Taipei, Taiwan

**Keywords:** Nanoparticle vaccine, SARS-CoV-2, COVID-19, durable antibody response, cross-protectivity, variants of concern

## Abstract

Numerous vaccines have been developed to address the current COVID-19 pandemic, but safety, cross-neutralizing efficacy, and long-term protectivity of currently approved vaccines are still important issues. In this study, we developed a subunit vaccine, ASD254, by using a nanoparticle vaccine platform to encapsulate the SARS-CoV-2 spike receptor-binding domain (RBD) protein. As compared with the aluminum-adjuvant RBD vaccine, ASD254 induced higher titers of RBD-specific antibodies and generated 10- to 30-fold more neutralizing antibodies. Mice vaccinated with ASD254 showed protective immune responses against SARS-CoV-2 challenge, with undetectable infectious viral loads and reduced typical lesions in lung. Besides, neutralizing antibodies in vaccinated mice lasted for at least one year and were effective against various SARS-CoV-2 variants of concern, including B.1.1.7 (Alpha), B.1.351 (Beta), P.1 (Gamma), B.1.617.2 (Delta), and B.1.1.529 (Omicron). Furthermore, particle size, polydispersity index, and zeta-potential of ASD254 remained stable after 8-month storage at 4°C. Thus, ASD254 is a promising nanoparticle vaccine with good immunogenicity and stability to be developed as an effective vaccine option in controlling upcoming waves of COVID-19.

## Introduction

At the end of 2019, a novel beta coronavirus, named severe acute respiratory syndrome coronavirus 2 (SARS-CoV-2), began to rapidly spread and infect human beings over the world. Since the official declaration of coronavirus disease 2019 (COVID-19) as a pandemic by the World Health Organization (WHO) on 11 March 2020, there have been over 593 million cases and more than 6.4 million deaths as of August 2022 (http://covid19.who.int). An effective vaccine is considered to be the solution to curtail this pandemic. To date, around 21 SARS-CoV-2 vaccines have been authorized for emergency use and 12 of them have been fully approved in at least one country. Though these vaccines demonstrated high efficacy with lower morbidity and mortality against SARS-CoV-2 infection in initial vaccination, neutralizing antibodies produced by the vaccines gradually decrease over time [1–3]. As the COVID-19 pandemic continues to spread around the world and new related variant-of-concern viral strains continue to appear, there is still a need to develop a next-generation COVID-19 vaccine of broader and more durable effect.

In this study, we adopted protein vaccine technology to develop a SARS-CoV-2 vaccine because of its safety profile and suitability for frequent use in prime-boost regimens [4]. The SARS-CoV-2 spike glycoprotein is a major component of the viral envelope that facilitates viral entry and serves as a major target of the host immune defense. In particular, the receptor-binding domain (RBD) of the spike protein mediates binding to the human angiotensin-converting enzyme 2 (hACE2) receptor and most of the immune-dominant epitopes for inducing neutralizing antibodies are located in the RBD. Thus, RBD is considered as a good immunogen for vaccine development [5–7]. Indeed, 90% of the neutralizing antibodies in COVID-19 convalescent patients were targeted to the RBD region [8], which indicated that it is the prime target to induce a protective immune response upon natural infection. In addition, as compared with the full-length spike protein, the higher yield of the RBD protein from cultured cells making it more suitable for large-scale production of vaccines. Animal and clinical trials have shown that immunization with RBD protein elicits neutralizing antibodies that can block SARS-CoV-2 binding to hACE2 [9–12].

Although RBD protein is a good vaccine target, its poor immunogenicity requires adjuvants to enhance the immune response [13, 14]. In this study, we used a nanoparticle platform, ASD25x, to deliver the RBD protein. ASD25x is a nanocomplex consisting of positively charged chitosan and negatively charged poly-γ-glutamic acid (γ-PGA). Chitosan is a natural polysaccharide with nontoxic, biocompatible, and biodegradable properties. It can enhance both humoral and cell-mediated immune responses [15]. γ-PGA is an amphiphilic polymer that can effectively deliver antigens to antigen-presenting cells [16]. Use of this nanoparticle vaccine platform to deliver antigens could generate high-titer IgG antibodies and T-cell responses in animal models [17, 18].

In this study, we generated the ASD254 vaccine by encapsulating the SARS-CoV-2 RBD in the ASD25x platform. ASD254 elicited potent antibody responses and effective protection to mice against SARS-CoV-2 challenge. Importantly, we found that neutralizing antibody titers can be sustained for at least 1-year post-vaccination and were able to cross-react with five SARS-CoV-2 variants of concern: B.1.1.7 (Alpha), B.1.351 (Beta), P.1 (Gamma), B.1.617.2 (Delta), and B.1.1.529 (Omicron). These results demonstrate that ASD254 can be a promising vaccine candidate against current and future SARS-CoV-2 mutant strains.

## Materials and methods

### Study approval

All mouse experiments were conducted in accordance with the “Guideline for the Care and Use of Laboratory Animals” as defined by the Council of Agriculture, Taiwan and was approved by the Institutional Animal Care and Use Committee of Academia Sinica (protocol ID: 20-05-1471 and 19-07-1330). The convalescent serum samples were collected from 12 participants who had COVID-19 as confirmed by RT-PCR and provided written informed consent with approval by the Institutional Review Board of Academia Sinica (AS-IRB-BM-20006). Experiments with infectious SARS-CoV-2 virus strains under BSL3 conditions were approved by Institutional Biosafety Committee of Academia Sinica. All sample processes were conducted according to the “Interim Laboratory Biosafety Guidelines for Handling and Processing Specimens Associated with Coronavirus Disease 2019 (COVID-19)” recommended by the Taiwan CDC.

### Animals

BALB/c and C57BL/6 mice were purchased from the National Laboratory Animal Center (Taipei) and maintained in a specific pathogen-free environment in the animal facilities of the Institute of Biomedical Sciences, Academia Sinica. All experimental procedures were reviewed and approved by the Animal Care and Use Committee of Academia Sinica.

### RBD construct design and recombinant protein production

The gene encoding Wuhan-Hu-1 isolate (GenBank YP_009724390.1) SARS-CoV-2 RBD (residues 319–541) was obtained from a previously established pUC57-nCOV-spike construct provided by Dr. Che Ma (Genomics Research Center, Academia Sinica, Taipei, Taiwan) and cloned into the pCDNA3.1 (Invitrogen, CA, USA) vector with the Igκ leader sequence (ATGGAGACAGACACACTCCTGCTATGGGTACTGCTGCTCTGGGTTCCAGGTTCCACCGGTGAC) at the 5′ end and the His6 tag sequence (CACCACCACCACCACCACAT) at the 3′end of the RBD fragment. The RBD-expressing plasmid was transfected into ExpiCHO cells (Thermo Fisher Scientific, MA, USA) for recombinant RBD protein production. Supernatants were collected and centrifuged to discard cellular debris 10 days post-transfection. ProBond Nikel-Chelating Resin column (Invitrogen, CA, USA) was used to purify His-tagged recombinant RBD protein. Concentration of the purified protein was measured by BCA assay (Thermo Fisher Scientific, MA, USA) and the purity was measured by SDS-PAGE (Invitrogen, CA, USA). Western blot analysis was used to identify the recombinant RBD protein with anti-RBD antibody (Sino Biological, Beijing) and anti-His antibody (COVANCE, NJ, USA).

### RBD binding assay

To measure the binding capacity of recombinant SARS-CoV-2 RBD protein with different receptors, 3T3 cells were transfected with hACE2 or hDPP4 using lipofectamine 2000 (Invitrogen, MA, USA). Two days post-transfection, cells were harvested and incubated with 1 μg SARS-CoV RBD protein (Sino Biological, Beijing), recombinant SARS-CoV-2 RBD protein, or MERS-CoV RBD protein (Sino Biological, Beijing) in 100 µl staining buffer (1% FBS/DPBS) for 1 h. After washing out the non-binding protein, cells were incubated with polyclonal anti-RBD antibody for 30 min. Cells were then washed with staining buffer again and incubated with 0.5 μg PE-conjugated goat anti-mouse IgG-Fc (Jackson Laboratory, ME, USA) in 100 μl staining buffer for 30 min. Cell viability dye, 7-amino-actinomycin D (7-AAD) (Biolegend, CA, USA), was used to exclude non-viable cells. Stained cells were analyzed by FACSCanto (BD Biosciences, NJ, USA) and data were processed using FlowJo V10 software (BD Biosciences, NJ, USA).

### Vaccine formulation

SARS-CoV-2 RBD nanoparticle vaccine encapsulation was performed as described [17] and the nanoparticle was named ASD254. Formulation is revealed with approval from ASCENDO Biotechnology (Taipei) as follows. Briefly, chitosan (HMC+, Halle, Germany) solution (w/v = 2.5% in 1% acetic acid) was mixed with 0.05M PB buffer before adding to 0.25 mg recombinant SARS-CoV-2 RBD and γ-PGA (HABIER, Shandong, China) pre-mixing solution (w/v = 1% in ddH_2_O) to form the SARS-CoV-2 RBD nanoparticle vaccine. Empty nanoparticle without loading protein was prepared as a control and called ASD25x. For quality control, the size, zeta-potential, polydispersity index (PdI), and the encapsulation efficiency (EE) of the nanoparticle vaccine were measured after production of every batch. Size, zeta-potential, and PdI of the nanoparticle vaccine were determined by Zetasizer Nano ZS (Malvern Panalytical, Malvern, WR, UK). To measure the encapsulation efficiency, the ASD254 nanoparticles were centrifuged in 100 kDa Amicon Ultra-0.5 centrifugal filter unit at 13,000 g for 6 min at room temperature. The flow-through was collected and the amount of free-form RBD protein was determined by measuring the fluorescent signal of the RBD protein (excitation: 280 nm/emission: 335 nm) using Varioskan^TM^ LUX multimode microplate reader (Thermo Fisher Scientific, MA, USA).

For aluminum-adjuvanted RBD vaccine (Alum-RBD), the recombinant SARS-CoV-2 RBD protein was diluted with PBS and mixed continuously with Imject Alum (Thermo Fisher Scientific, MA, USA) at a 1:1 ratio for 1 h before injection.

### Transmission electron microscopy

An amount of 10 μl ASD254 was placed on a formvar/carbon-coated grid and blotted with filter paper 3 times. The grid was then stained with 10 µl of 1% phosphotungstic acid solution for 1 min and then blotted 3 times. The stained specimen was dried under room temperature and 5 in-Hg conditions. The grid was observed under an H7500 transmission electron microscope (Hitachi, Tokyo) at an acceleration voltage of 80 keV.

### Mouse experiments

For immunization, 6- to 8-week-old female C57BL/6 or BALB/c mice were subcutaneously immunized with ASD25x, ASD254 (3, 5, or 10 µg as described in the text), or Alum-RBD vaccine (10 µg) on days 0, 14, and 28. Serum samples were collected 2 weeks after each immunization or at indicated time points. For dosage experiment, 6- to 8-week-old female C57BL/6 or BALB/c mice were subcutaneously immunized with 10 µg of ASD254 on days 0, and 14, or 20 µg of ASD254 on day 0. Serum samples were collected on day 42.

For SARS-CoV-2 challenge, mice were anesthetized and transduced with 3 × 10^11^ vg AAV6/hACE2 intratracheally and 1 × 10^12^ vg AAV9/hACE2 intraperitoneally at day 56 after the first immunization [19]. The transduced mice were then challenged with 8 × 10^4^ TCID_50_ of SARS-CoV-2 (wild-type, hCoV-19/Taiwan/4/2020) intranasally. Mouse body weight was monitored daily. Five days post-challenge, mouse lungs were harvested for viral load quantification and lung pathophysiology analysis. All animal experiments with SARS-CoV-2 challenge were conducted in an animal biosafety level 3 (ABSL3) facility in the Genomics Research Center, Academia Sinica (Taipei, Taiwan).

### SARS-CoV-2 RBD-specific total IgG ELISA

Ninety-six-well plates (Thermo Fisher Scientific, MA, USA) were coated with 5 µg/ml RBD protein at 4°C overnight. Plates were blocked with 3% skim-milk/PBS at room temperature for 2 h. Serum samples were serially diluted and added to the blocked plates before incubation at room temperature for 1 h. After incubation, bound antibodies were detected with goat anti-mouse IgG-Fc HRP-conjugated antibody (Chemicon, CA, USA). TMB substrate (BD Biosciences, NJ, USA) was added into the plates and peroxidase reactions were stopped by adding 2N H_2_SO_4_. The absorbance at 450 nm was measured using the EMax Microplate reader (Molecular Devices, CA, USA). The endpoint dilution titer was determined when the titer value of the last serum dilution was 2-fold above the blank value.

### SARS-CoV-2 pseudovirus neutralization assay

293 T cells that stably expressed human ACE2 (293T-hACE2) and lentiviral-based pseudotyped SARS-CoV-2 viruses were provided by the National RNAi Core Facility (Academia Sinica, Taipei, Taiwan). One day before neutralization assay, 293T-hACE2 cells were seeded into 96-well black plates (Perkin Elmer, MA, USA) at 1 × 10^4^ cells per well at 37°C. Mouse serum was inactivated at 56°C for 30 min and serially diluted by 4-fold with culture medium before incubation with the indicated SARS-CoV-2 pseudovirus for 1 h. The mixtures were then added to pre-seeded 293T-hACE2 cells and incubated for 3 days. Luciferase activity was measured using the Luciferase Assay kit (Promega, WI, USA). The 50% neutralization titer (NT_50_) was calculated using nonlinear regression with Prism software 8.1.0 (GraphPad Software Inc.).

### Authentic SARS-CoV-2 neutralization assay

Wild-type SARS-CoV-2 virus (hCoV-19/Taiwan/4/2020) was used for live virus micro-neutralization assay and experiments were performed in an approved biosafety level 3 (BSL-3) facility. Mouse serum was inactivated at 56°C for 30 min, serially diluted by 2-fold, and incubated with 100 TCID_50_ SARS-CoV-2 virus for 1 h. The mixtures were then added to Vero-E6 cells for 4-day incubation. Cells were then fixed with 10% formaldehyde and stained with 0.5% crystal violate for 20 min. The plates were washed with distilled water and scored for infection. The 50% neutralizing titers were calculated by the Reed and Muench Method.

### SARS-CoV-2 viral RNA quantification

The level of SARS-CoV-2 viral RNA in challenged mice was quantified as described [19]. Briefly, lung tissues of challenged mice were weighed and homogenized by SpeedMill PLUS (Analytik Jena AG, Jena, Germany) and total RNA was extracted using the RNeasy Mini kit (QIAGEN, Hilden, Germany) following the manufacturer’s instruction. SARS-CoV-2 viral RNA was quantified using the Superscript III one-step RT-PCR TaqMan assay with Platinum Taq Polymerase (Thermo Fisher Scientific, MA, USA) by the Applied Biosystems 7500 Real-Time PCR System (Thermo Fisher Scientific, MA, USA). A synthetic 113-bp oligonucleotide fragment (synthesized by Genomics BioSci and Tech, Taipei, Taiwan) was used as the standard to estimate copy numbers of the viral genome.

### SARS-CoV-2 tissue culture infectious dose assay

Lung tissues of challenged mice were weighed and homogenized in 1 ml DMEM with 2% FBS and 1% P/S by SpeedMill PLUS (Analytik Jena AG, Jena, Germany) and clarified by centrifugation at 13,000 rpm for 10 min at 4°C. Infectious viral titers were determined by incubating 10-fold serially diluted samples with Vero-E6 cells in quadruplicate and cytopathic effects (CPEs) were observed after 4 days. Viral titer was expressed as 50% tissue culture infectious dose (TCID_50_)/ml, which was calculated by the Reed and Muench method.

### Histopathological examination and immunofluorescence staining

Lungs from challenged mice were fixed with 4% paraformaldehyde before being embedded in paraffin. For analysis of lung pathophysiology, tissue sections were deparaffinized, rehydrated, and stained with hematoxylin and eosin (H&E). Images were captured by Pannoramic 250 FLASH II digital slide scanner (3DHistech, Hungary). The pathophysiology was evaluated according to a histopathological scoring system as described [20, 21].

For detecting SARS-CoV-2 nucleocapsid (N) protein expression, tissue sections were deparaffinized, rehydrated, and heated in citrate buffer (pH 6.0) for antigen retrieval. After cooling, sections were washed with PBS twice and permeabilized with 0.2% Tween-20/PBS for 20 min. 5% goat serum in PBS was used to block nonspecific binding, and human anti-SARS-CoV-2 N protein antibody (provided by Dr. An-Suei Yang, Genomics Research Center, Academia Sinica, Taipei, Taiwan) was used to detect intracellular viral N protein expression. After incubation, tissue sections were stained with Alexa Fluor 568- conjugated goat anti-human IgG (Invitrogen, CA, USA) at 1:1000 dilution for 1 h, counterstained with DAPI (Invitrogen, CA, USA), and mounted with fluorescence mounting medium (Vector Laboratories, CA, USA). Slide imaging was done using a Zeiss LSM700 Stage confocal microscope (Carl Zeiss Microscopy GmbH, Jena, Germany).

### Statistical analysis

Results are presented as mean ± SD. One-way ANOVA with Tukey’s comparison was used to analyze differences across multiple groups of animals. Two-way ANOVA with Sidak’s multiple comparisons test was used to analyze differences in body weight between SARS-CoV-2-challenged ASD25x- and ASD254-vaccinated mice. Unpaired *t* test was used to analyze differences between the two experimental groups of animals. *P* < 0.05 was considered to be statistically significant.

## Results

### Production and characterization of SARS-CoV-2 RBD protein and ASD254 nanoparticles

**Figure 1.**
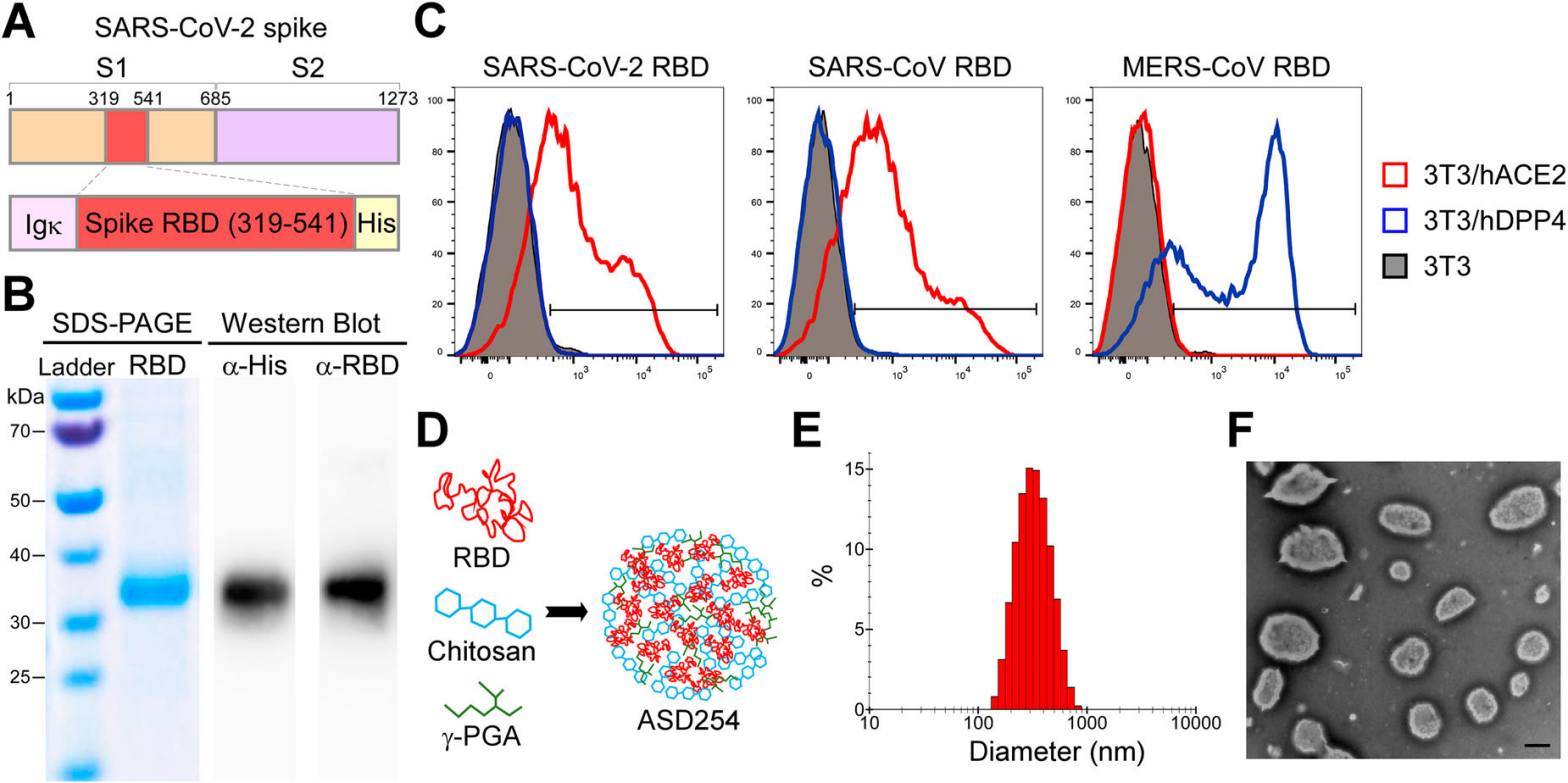
Construction and characterization of receptor-binding domain (RBD) protein and ASD254 nanoparticle vaccine. (A) Diagrammatic illustration of the SARS-CoV-2 spike protein and the recombinant RBD-His protein. The recombinant RBD protein consists of residues 319–541 of the SARS-CoV-2 spike protein of the Wuhan-Hu-1 isolate. The Igκ leader was added to the N-terminus of the RBD and a His6 tag was added to the C-terminus. (B) Validation of purified recombinant RBD protein. An amount of 3 μg of the purified recombinant RBD protein was used for SDS-PAGE and western blot analysis, with anti-RBD and anti-His antibody staining the purified recombinant RBD protein. (C) Flow cytometry of purified recombinant RBD binding against hACE2 and hDPP4. Purified recombinant RBD protein was incubated with parental, hACE2- or hDPP4-expressing 3T3 cells to test its binding capacity. The red line shows the binding of recombinant RBD protein with hACE2, the blue line shows the binding with hDPP4 and the gray shading shows the binding with parental 3T3 cells. (D) Diagram of the ASD254 nanoparticle and its composition. (E) Size distribution of ASD254 particles. (F) TEM image of ASD254. Scale bar, 500 nm. See also Figure S1.

To use the SARS-CoV-2 spike receptor-binding domain (RBD) protein as an immunogen, we modified the original RBD genome sequence to increase *in vitro* expression efficiency and gain the ability to secrete from cells. We constructed the codon-optimized RBD (residues 319-541) and fused with an Igκ signal peptide and 6x His tag for protein secretion and purification, respectively (Figure 1A). The ExpiCHO system (Thermo Fisher Scientific, MA, USA) was used to express the recombinant RBD protein to ensure proper folding and glycosylation. The purified recombinant RBD protein could be recognized by both anti-His and anti-RBD antibodies (Figure 1B); nevertheless, its molecular weight shifted from the predicted 27 kDa to > 30 kDa, probably because of the dense glycosylation of RBD protein [9].

To further determine the integrity of the purified recombinant RBD protein, we assessed its hACE2 binding capacity by flow cytometry. The SARS-CoV RBD protein (Sino Biological, Beijing), which also binds to hACE2 [22], and MERS-CoV RBD protein (Sino Biological, Beijing), which binds to human dipeptidyl peptidase 4 (hDPP4) [23], were included as controls. 3T3 cells were transiently transfected with hACE2 or hDPP4 for 2 days, then co-cultured with each RBD protein. Recombinant SARS-CoV-2 RBD protein and SARS-CoV RBD protein selectively bound to 3T3 cells expressing hACE2 but not parental or hDPP4-expressing 3T3 cells, and MERS-CoV RBD protein bound to hDPP4-expressing 3T3 cells exclusively (Figure 1C). These results showed that our recombinant SARS-CoV-2 RBD protein was successfully constructed, expressed, and purified with the expected binding ability to hACE2, which indicates the proper conformation of this protein.

Next, to promote immunogenicity, the purified recombinant SARS-CoV-2 RBD protein was encapsulated with charged polymers chitosan and poly-γ-glutamic acid (γ-PGA) (Figure 1D), with encapsulation rate > 84% (Figure S1A). The encapsulated recombinant SARS-CoV-2 RBD, hereafter named ASD254, was generated as a stable, dispersive spherical nanoparticle with positive charge (Figure S1A). The average size was about 340 nm in diameter (Figures 1E and S1A). Transmission electron microscopy was used to visualize and confirm the presence of intact ASD254 nanoparticles with the expected size (Figure 1F). Besides, ASD254 nanoparticles produced in every lot showed good consistency (Figure S1B). To examine the stability of ASD254, the basic properties of the ASD254 nanoparticle, including particle size, polydispersity index (PdI), and zeta-potential, were measured before and after long-term storage. After 8-month storage at 4°C, the monitored properties of ASD254 remained comparable to that of the initial preparation (Figure S1C). Hence, ASD25x can be used to encapsulate the recombinant SARS-CoV-2 RBD protein to form a stable nanoparticle vaccine for SARS-CoV-2.

### Immunization with ASD254 elicits potent neutralizing antibody response in mice

**Figure 2.**
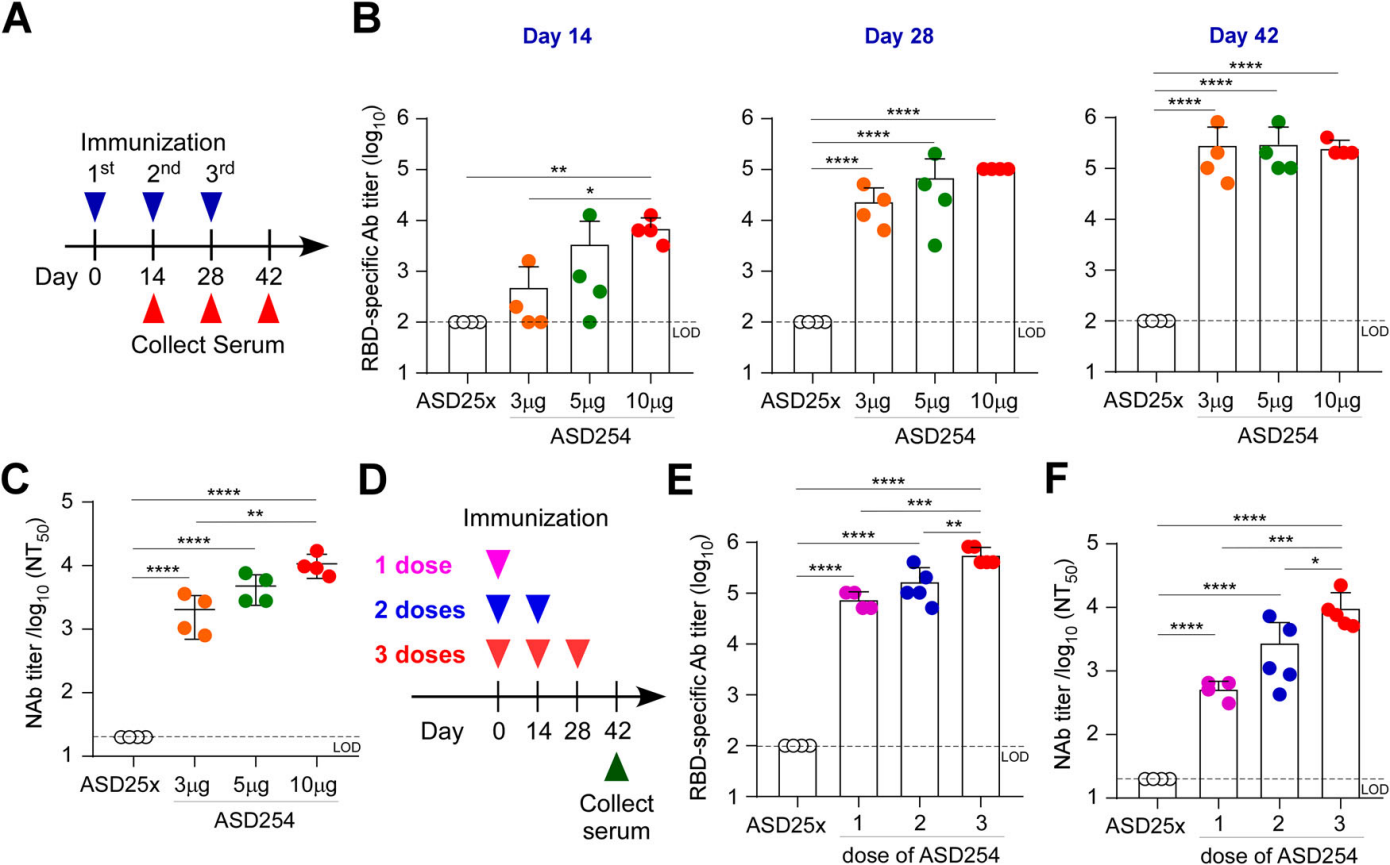
ASD254 vaccine elicits RBD-specific antibody response dose-dependently. (A) Immunization and blood draw schedule. BALB/c mice were subcutaneously vaccinated with ASD25x (*n* = 4) or ASD254 (*n* = 4 of each group) of 3, 5, or 10 μg on days 0, 14, 28. Mouse serum was collected 2 weeks after every vaccination. (B) Serum IgG binding to recombinant SARS-CoV-2 RBD measured by ELISA. (C) Serum neutralizing activity against SARS-CoV-2 pseudovirus measured by neutralization assay. (D) Immunization and blood draw schedule. BALB/c mice were subcutaneously vaccinated with ASD25x (*n* = 4), or 1 dose (*n* = 4), 2 doses (*n* = 5) or 3 doses (*n* = 5) of ASD254. (E) Serum IgG binding to recombinant SARS-CoV-2 RBD measured by ELISA. (F) Serum neutralizing activity against SARS-CoV-2 pseudovirus measured by neutralization assay. The dotted line indicates the limit of detection. Differences among all tested groups were analyzed by one-way ANOVA with multiple comparisons test. *p < 0.05, **p < 0.01, ***p < 0.001, ****p < 0.0001. See also Figure S2.

To assess the immunogenicity of ASD254 as a vaccine, BALB/c mice were subcutaneously vaccinated with 3, 5 and 10 μg of ASD254 in a prime-boost schedule on days 0, 14, and 28, and were monitored for antibody responses every 2 weeks after the primary vaccination (Figure 2A). The empty nanoparticle (ASD25x) was included as a control. Though ASD254-vaccinated mice showed lower titers of RBD-specific IgG antibodies when immunized with 3 or 5 μg than 10 μg (mean endpoint titers of 7.2 × 10^3^ with 10 μg, 3.5 × 10^3^ with 5 μg, 5.0 × 10^2^ with 3 μg, Figure 2B) after the first vaccination, lower dose of ASD254 could still elicit a relatively potent antibody response after the second vaccination, and reached comparable RBD-specific antibody titers as the 10-μg–vaccinated group after the third vaccination (Figure 2B). To verify the neutralizing activity of vaccine-elicited antibodies in immunized mice, neutralization assay using lentiviral-based SARS-CoV-2 pseudovirus was performed. Serum collected on day 42 from ASD254-vaccinated mice of every dose neutralized pseudotyped SARS-CoV-2 virus in a dose-dependent manner (Figure 2C). Similar results were shown in experiments with the C57BL/6 mouse model, where a lower dose of ASD254 induced RBD-specific antibody responses and elicited potent neutralizing antibody responses after boosting (Figures S2A and S2B). Collectively, we demonstrated that ASD254 vaccine could stimulate potent neutralizing antibody responses in a dose-dependent fashion and reached comparable RBD-specific antibody titers after boosting even with lower doses.

As currently available vaccines are given in two doses in primary series, we assessed whether two doses of ASD254 were sufficient to induce neutralizing antibody responses. In addition, a single higher dose (20 μg) of ASD254 was also exanimated. BALB/c mice were subcutaneously vaccinated with a single high dose (20 μg) of ASD254 on day 0, two doses (10 μg per dose) on days 0 and 14, or three doses (10 μg per dose) on days 0, 14 and 28. Sera from vaccinated mice were collected on day 42 after the first immunization to assess RBD-specific IgG and neutralizing antibodies (Figures 2D). Both single and two shots of ASD254 were sufficient to induce an RBD-specific antibody response with potent neutralizing activity (Figure 2E and F) without significant differences. However, the three-dose regimen was still the most efficient way to elicit neutralizing antibody responses. Similar results were demonstrated in the C57BL/6 mouse model (Figure S2C). Taken together, a single high dose or two doses of ASD254 vaccination was sufficient to induce potent titer of RBD-specific IgG, but a three-dose regimen was still the most optimal approach to elicit high neutralization antibody response.

**Figure 3.**
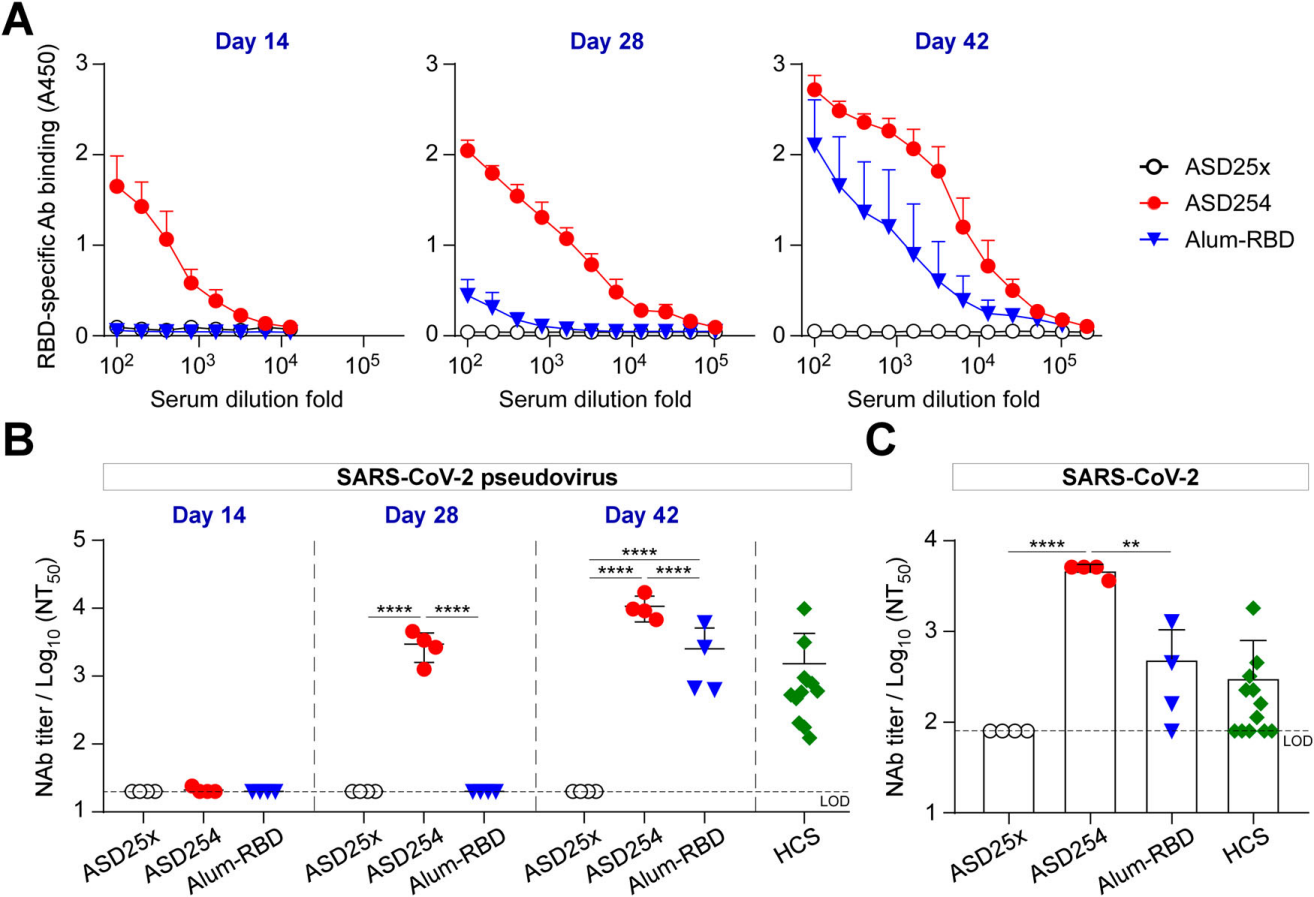
ASD254 vaccine elicits potent neutralizing antibody response against SARS-CoV-2 infection. BALB/c mice were subcutaneously immunized with ASD25x (*n* = 4), ASD254 (*n* = 4) or Alum-RBD (*n* = 4) on days 0, 14, and 28. Mouse serum was collected 2 weeks after every immunization. (A) Serum IgG binding to recombinant SARS-CoV-2 RBD measured by ELISA. (B) Serum neutralizing activity against SARS-CoV-2 pseudovirus measured by neutralization assay. HCS, COVID-19 human convalescent sera. (C) Serum neutralizing activity against SARS-CoV-2 measured by live virus micro-neutralization assay. The dotted line indicates the limit of detection. Differences between groups were calculated by one-way ANOVA with multiple comparisons test. *p < 0.05, **p < 0.01, ***p < 0.001, ****p < 0.0001. See also Figure S3.

Next, we compared the immunogenicity of ASD254 with aluminum-adjuvant RBD vaccine (Alum-RBD) as aluminum is a conventional and most commonly used adjuvant in licensed human vaccines since 1920s [24, 25]. BALB/c mice were subcutaneously vaccinated with 10 μg ASD254, Alum-RBD or ASD25x in a prime-boost schedule on days 0, 14, and 28. Sera from vaccinated mice were collected every 2 weeks post-vaccination for monitoring the RBD-specific IgG production. Fourteen days after the first vaccination, ASD254 nanoparticle elicited RBD-specific antibody responses, with mean endpoint titer of 7.2 × 10^3^, whereas Alum-RBD did not induce detectable RBD-specific antibody production (Figure 3A). After the second immunization, ASD254-vaccinated mice showed enhanced RBD-specific antibody titers (mean endpoint titer of 1.0 × 10^5^) on day 28. In contrast, Alum-RBD-vaccinated mice showed detectable titers of RBD-specific antibodies (mean endpoint titers = 9.0 × 10^2^) which was hundred-fold lower than that of ASD254-vaccinated mice (Figure 3A). After the third vaccination, although both ASD254 and Alum-RBD vaccination induced potent RBD-specific antibody responses with mean endpoint titers of 2.6 × 10^5^ in ASD254 mice and 5.4 × 10^4^ in Alum-RBD mice, the RBD-specific antibody titers were about 4.8-fold higher in ASD254- than Alum-RBD-vaccinated mice on day 42 (Figure 3A). Similar results were found in the C57BL/6 mouse model, where ASD254 induced higher titers and an earlier response of RBD-specific antibodies than Alum-RBD (Figure S3A). These data indicated that encapsulating SARS-CoV-2 RBD protein into the ASD25x nanoparticle platform could largely improve the immunogenicity of RBD protein and stimulated a potent RBD-specific antibody response in both BALB/c and C57BL/6 mouse models.

Neutralization assay using pseudovirus was also conducted to evaluate the neutralizing activity of vaccine-elicited antibodies in immunized mice. In BALB/c mice, the ASD254 nanoparticle but not Alum-RBD elicited serum-neutralizing antibodies after the second immunization on day 28, with mean 50% neutralizing titer (NT_50_) of 1.0 × 10^3^ and reached a higher NT_50_ after the third vaccination on day 42 (mean NT_50_ = 1.1 × 10^4^) (Figure 3B). These levels of NT_50_ matched or exceeded those of a panel of 12 COVID-19 human convalescent serum (HCS) (mean NT_50_ of 1.5 × 10^3^). By contrast, neutralizing antibodies could be detected in Alum-RBD-vaccinated mice after the third vaccination on day 42, with titers much lower than that by ASD254 (mean NT_50_ of 2.5 × 10^3^, Figure 3B). This result was verified by neutralization assay using authentic SARS-CoV-2 on serum collected from vaccinated mice after the third vaccination on day 42. The mean NT_50_ of ASD254-vaccinated mouse serum was 9-fold higher than both Alum-RBD-vaccinated mouse serum and COVID-19 HCS (Figure 3C). Similar results were also shown in the C57BL/6 mice immunized with ASD254, with mean NT_50_ of 5.0 × 10^3^ in pseudovirus and 2.3 × 10^3^ in live virus neutralization assays after the third vaccination on day 42 (Figure S3B and S3C). In contrast to the BALB/c mouse model, Alum-RBD failed to induce neutralizing antibody responses in the C57BL/6 mouse model even after the third vaccination on day 42 (Figure S3B and S3C). These results indicated that ASD254 performed much better than conventional adjuvant mixed Alum-RBD in inducing high titers of neutralizing antibodies against SARS-CoV-2.

### ASD254 vaccine elicits SARS-CoV-2 RBD-specific T-cell response and relatively balanced T helper 1 (Th1) and Th2-cell immune responses

Besides evaluating humoral immune responses, we also evaluated whether ASD254 could stimulate cellular immune responses. Splenocytes from vaccinated mice were isolated and stimulated with RBD protein for 3 days. As compared to the ASD25x control group, splenocytes from ASD254- and Alum-RBD-vaccinated mice rapidly proliferated in response to RBD stimulation (Figure S4A). Besides, an increment of IFN-γ secreting CD8^+^ T cells were also observed upon RBD stimulation in ASD254- and Alum-RBD-vaccinated mice (Figure S4B). Thus, both ASD254 and Alum-RBD vaccines could elicit RBD-specific cellular immune responses against SARS-CoV-2. Differentiation of Th cells was also evaluated. Th cells can be distinguished by their cytokine production patterns, with Th1 cells producing IFN-γ and IL-2, and Th2-cells producing IL-4 [26], so we assessed cytokine composition in cultured cell supernatants of ASD254- and Alum-RBD-vaccinated mouse splenocytes after 3 days of *ex vivo* RBD stimulation. Level of Th1 cell-related cytokines was significantly increased in response to RBD protein stimulation in ASD254-vaccinated mice (Figures S4C and S4D). The Th2-cell-related cytokine level was also increased in response to RBD protein stimulation in ASD254-vaccinated mice but was substantially lower than the Alum-RBD group, which was considered to induce a Th2-cell dominant immune response (Figure S4E). These results demonstrated that vaccination with ASD254 induced a relatively balanced Th1- and Th2-cell immune responses, whereas vaccination with Alum-RBD resulted in a Th2-cell-biased immune response.

### ASD254 protects mice against SARS-CoV-2 infection

**Figure 4.**
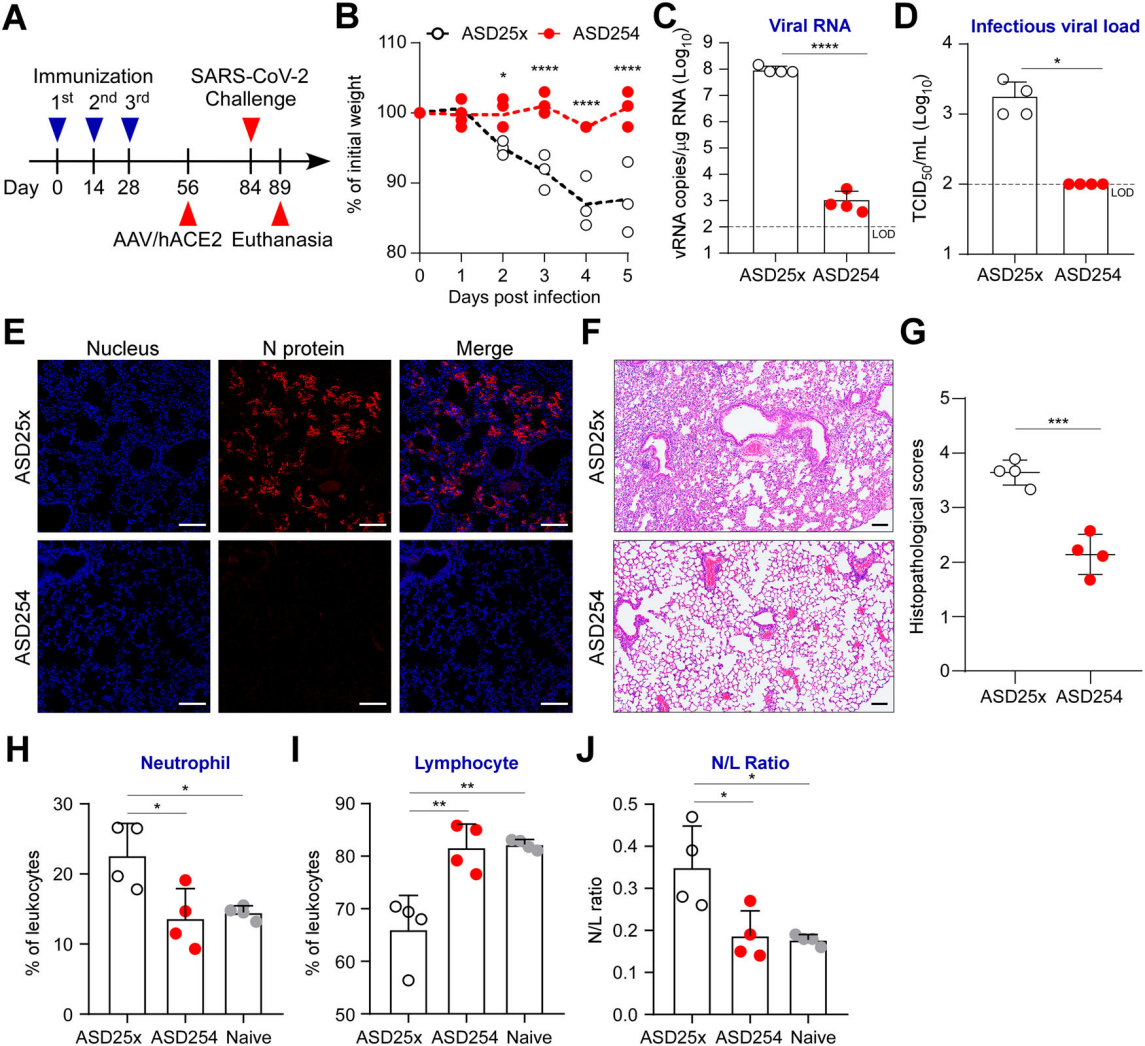
ASD254 protects mice against SARS-CoV-2 infection. (A) Immunization, AAV/hACE2 transduction, and SARS-CoV-2 challenge schedule. One month after priming and boosting with ASD254 (*n* = 4) or ASD25x (*n* = 4), immunized BALB/c mice were transduced with AAV/hACE2 by intratracheal and intraperitoneal administration. One month later, animals were challenged with 8 × 10^4^ PFU SARS-CoV-2 intranasally. (B) Body weight change of SARS-CoV-2–challenged mice. (C and D) Viral genomic RNA copies (C) and infectious viral load (D) in lung of SARS-CoV-2–challenged mice measured by RT-qPCR and TCID_50_ assay. The dotted line indicates the limit of detection. (E) Viral N protein expression in lungs of SARS-CoV-2–challenged mice. Red, N protein; blue, nucleus. Scale bar, 100 μm. (F) H&E staining for lung pathophysiology of SARS-CoV-2-challenged mice. Scale bar, 100 μm. (G) Histopathological lung scores in SARS-CoV-2-challenged mice. (H and I) Complete blood count composition change of peripheral neutrophils (H) and lymphocytes (I) in naïve or SARS-CoV-2-challenged mice. (J) Ratio of neutrophils to leukocytes (N/L) of naïve or SARS-CoV-2-challenged mice. Differences between groups were calculated by unpaired *t* test, one-way or two-way ANOVA with multiple comparisons test. *p < 0.05, **p < 0.01, ***p < 0.001, ****p < 0.0001.

To further assess the protection efficacy of ASD254 vaccine against SARS-CoV-2 *in vivo*, we conducted a SARS-CoV-2 challenge experiment with adeno-associated virus (AAV)/hACE2 mice, a model displaying severe COVID-19 which was generated by transducing mice with AAV encoding hACE2 to enable viral receptor expression and SARS-CoV-2 infection in lung [19]. BALB/c mice were vaccinated with ASD254 on days 0, 14 and 28 and transduced with AAV/hACE2 on day 56 (Figure 4A). One month after transduction, mice were challenged with SARS-CoV-2 and weighed daily. Mice vaccinated with ASD254 were protected against weight loss as compared with the ASD25x control group over a 5-day period (Figure 4B). Pulmonary viral loads were analyzed on day 5 post-challenge. ASD25x control mice had a mean of 9.8 × 10^7^ copies/μg total RNA, whereas ASD254-vaccinated mice had only limited detectable viral RNA copies in lung (1.1 × 10^3^ copies/μg total RNA, Figure 4C). Also, infectious virions generated during infection were measured. The viral titers reached a mean of 1.8 × 10^3^ tissue culture infectious dose (TCID_50_)/ml in ASD25x control mice, whereas TCID_50_ in ASD254-vaccinated mice were below the detection limit (Figure 4D). We used viral nucleocapsid (N) protein immunofluorescence staining to inspect the infection status in lung. We observed a large number of infected cells in lung sections of ASD25x control mice, but cells positive for N protein were not detectable in lung sections of ASD254-vaccinated mice (Figure 4E).

We also evaluated whether ASD254 vaccine could prevent lung pathologic and immunologic changes caused by the viral infection. Five days after SARS-CoV-2 challenge, histopathological examination of the lungs was conducted to determine whether challenged mice had pneumonia. Histopathological scores, which are used to evaluate the severity of inflammation and diffuse alveolar damage, were used to quantify the pathophysiological condition of the lung. ASD25x control mice showed typical lung lesions, characterized by severe septa thickening, alveoli shrinking, inflammatory cell infiltrating, and epithelial necrosis, whereas ASD254-vaccinated lung sections showed only little to mild pathological changes (Figure 4F and G). We assessed the composition change of neutrophils and lymphocytes in the peripheral blood of challenged mice as neutrophil-to-lymphocyte ratio (NLR) is one of the markers of subclinical inflammation and is positively correlated with disease severity in COVID-19 [27, 28]. Five days after challenge, mice vaccinated with ASD254 showed maintained NLR and prevented increase of neutrophils and decrease of lymphocytes (Figure 4H–J). Collectively, these results demonstrated that immunization with ASD254 could limit SARS-CoV-2 infection and the relevant pathological and immunological changes caused by the viral infection.

### Immunization with ASD254 generates durable neutralizing antibody responses and cross-protection activity against SARS-CoV-2 variants

**Figure 5.**
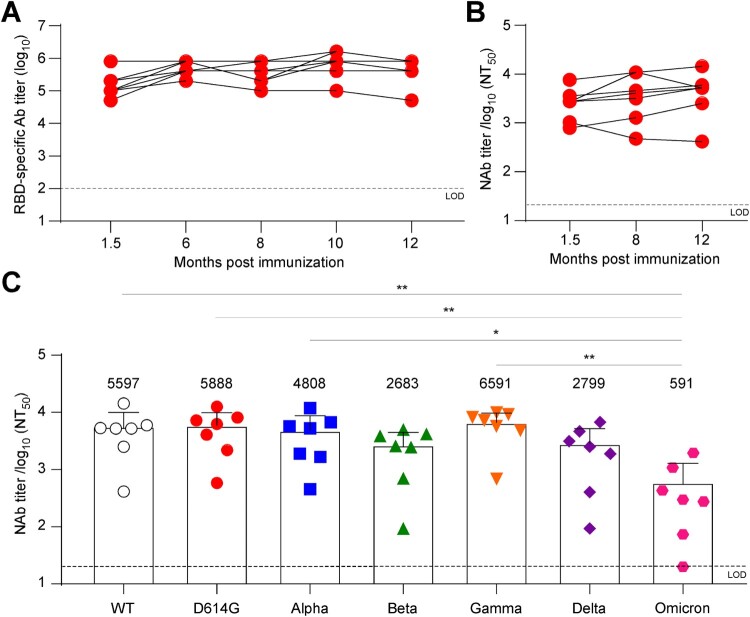
ASD254 vaccine induces durable and cross-variant neutralizing antibody responses. BALB/c mice were subcutaneously vaccinated with 5 µg (*n* = 3) or 3 µg (*n* = 4) ASD254 on days 0, 14, and 28. Mouse serum was collected at indicated time points. (A) Serum IgG binding to recombinant SARS-CoV-2 RBD measured by ELISA. (B) Serum neutralizing activity against wild-type SARS-CoV-2 pseudovirus measured by neutralization assay. (C) Serum samples were collected 12 months after immunization and serum neutralizing activity against wild-type, D614G, Alpha, Beta, Gamma, Delta, and Omicron SARS-CoV-2 pseudovirus was measured by neutralization assay. The dotted line indicates the limit of detection. Differences among all tested groups were analyzed by one-way ANOVA with multiple comparisons test. *p < 0.05, **p < 0.01, ***p < 0.001, ****p < 0.0001.

Besides immunogenicity, longevity and cross-neutralizing activity of vaccine-induced immunity are also critical issues for vaccine development. To address this, we monitored RBD-specific antibody responses in ASD254-vaccinated mice for 1 year. Serum was collected and evaluated for RBD-specific antibody titers at 1.5, 6, 8, 10 and 12 months after ASD254 immunization. Encouragingly, the RBD-specific antibodies were maintained at a high level without any significant decrease during the year (mean endpoint titers of 4.2 × 10^5^, Figure 5A). Also, serum collected from ASD254-vaccinated mice at 1.5, 8 and 12 months all neutralized pseudotyped SARS-CoV-2 virus efficiently and showed similar neutralizing titers over time (Figure 5B). These results indicated that immunization with ASD254 generated durable antibody responses that could last for at least a year without decline in a mouse model.

With the constant emergence of new circulating variants carrying several dominant mutations in the spike protein being identified, the efficacy of a vaccine based on the original strain has become a crucial issue. The D614G mutant became the dominant strain early in the pandemic and showed a fitness advantage because all emerging variants harbored this mutation [29]. The B.1.1.7 (Alpha; first identified in the United Kingdom), B.1.351 (Beta; first identified in South Africa), P.1 (Gamma; first identified in Brazil), B.1.617.2 (Delta; first identified in India), and B.1.1.529 (Omicron; first identified in South Africa) were designated as variants of concern with enhanced replication capacity, transmissibility and immunological escape [30]. To assess whether the ASD254 vaccine possesses cross-neutralizing activity, we evaluated the neutralizing titers of vaccinated mouse serum against these SARS-CoV-2 variants. Vaccinated mouse serum collected 12 months after vaccination was used to neutralize variant viruses. The D614G, Alpha, and Gamma variants showed no significant reduction in neutralizing titers as compared with the neutralizing activity of the original strain (wild-type [WT]) (Figure 5C). Despite an approximate 2.1-fold decrease against the Beta variant, a 2.0-fold decrease against the Delta variant, and a 9.5-fold decrease against the Omicron variant in neutralizing activity as compared with the WT were seen, ASD254-vaccinated mouse serum could still neutralize the virus efficiently (Figure 5C). These data indicated that ASD254 could generate durable neutralizing antibody responses that efficiently neutralized the WT and variant viruses for at least 1 year.

## Discussion

Since the outbreak of COVID-19 in late 2019, more than 593 million people had become infected and 6.4 million died worldwide (https://covid19.who.int). Developing a safe and effective vaccine is the current top priority. In this study, we developed an RBD-based nanoparticle vaccine against SARS-CoV-2 and demonstrated that vaccination with ASD254 induced potent neutralizing antibody responses and provided sufficient protection against SARS-CoV-2 challenges in AAV/hACE2-transduced mice. Additionally, ASD254 could induce durable neutralizing antibody titers for at least 1 year and provide cross-protection against SARS-CoV-2 variants of concern.

Several vaccines have been approved or authorized for emergency use in COVID-19. The Pfizer/BioNTech and Moderna mRNA vaccines were developed at a rapid pace and are > 90% effective [31, 32]. However, both vaccines require ultra-low-temperature storage (−20°C) and delivery, which greatly increases the cost and limits global distribution [33, 34]. Furthermore, these mRNA vaccines were authorized for use prophylactically in humans for the first time, and the anaphylaxis and long-term safety profile remain to be determined. The Oxford/AstraZeneca and Janssen/Johnson & Johnson adenoviral-based vaccines were also developed rapidly and can be transported at normal refrigerated temperature of 2–8°C [35, 36]. However, the nature of viral vector-induced anti-viral vector antibodies limited subsequent use of the same viral vector. Additionally, reports of rare events of thrombotic development after vaccination raised intensive safety concerns. To this end, although a protein-based vaccine is developed at a slower speed, it still shows potential as an effective and easily accessible vaccine. First of all, the protein subunit vaccine is a well-established technology that has been widely used for decades with an excellent track record for safety and effectiveness [4]. Second, it is easy to manufacture and relatively stable for storage and transportation, so the protein subunit vaccines are more ideal and accessible for global usage. Third, it could be used in a prime-boost approach to elicit more desired and prolonged immune responses. To date, several protein subunit vaccines have been approved or authorized for emergency use. NVX-CoV2373 from Novavax is considered to be one of the leading protein subunit vaccine for SARS-CoV-2, which has been approved for emergency use in 19 countries. It is a nanoparticle vaccine formed by inserting transmembrane domain of stabilized spike protein in micellar core of polysorbate 80 and adjuvanted with Matrix-M [37, 38]. Though struggling in manufacturing in the initial stage of development, NVX-CoV2373 has been reported to confer 89.7% protection against SARS-CoV-2 infection [39].

Previous study has demonstrated that an aluminum-adjuvant RBD vaccine could induce potent functional antibody responses and provide protection against SARS-CoV-2 challenge in mice and non-human primates (*Macaca mulatta*) without any detectable viral RNA or significant histopathological changes in lung [9]. In this study, as compared to the aluminum-adjuvant RBD vaccine, the RBD-encapsulated ASD25x vaccine showed enhanced immunogenicity and neutralizing antibody responses more potently and efficiently in both BALB/c and C57BL/6 mouse models. This encouraging data shows that ASD254 can be a vaccine candidate with high efficacy. Several recently published studies also showed superior immune responses when encapsulating RBD protein into different nanoparticles. The ferritin-based RBD nanoparticle vaccine elicited more robust neutralizing antibodies and cellular immune responses than the monomer vaccine and conferred protection against SARS-CoV-2 challenge in both hACE2 transgenic mice and rhesus monkeys [40–42]. The computationally designed I53-50 nanoparticle, which conjugates RBD protein into a trimeric form, showed 10-fold higher neutralizing antibody titers than a prefusion-stabilized spike trimer vaccine as well as promoted protective immunity against SARS-CoV-2 challenge in mice and non-human primates. The vaccine is now under evaluation in phase I/II clinical trials (NCT04742738 and NCT04750343) [43, 44]. Other RBD-conjugating nanoparticles such as AP205 capsid-like particles and mi3 synthetic virus-like particles also showed promising neutralization activity after immunization in mice or pigs [45, 46]. These studies support the use of nanoparticle vaccines as a potential alternative that can be both effective and stable. As compared with the aforementioned protein-based nanoparticles, which have stringent processes to remove endotoxin and microbial contamination during manufacture and higher cost, the non-protein-based nanoparticle used in our study is suitable for large-scale manufacture with lower cost [47]. Furthermore, protein-based nanoparticles need to fuse RBD with a particle scaffold or an additional Tag and Catcher added, whereas no additional modification of the RBD protein is needed when formulated into nanoparticles in our system. Thus, the ASD25x nanoparticle platform is more convenient and flexible in new vaccine design and manufacture.

Recently, the emergence of SARS-CoV-2 variants that appeared to be more infectious or caused more severe disease raised global concern [48–51]. Currently available vaccines are developed based on the sequence of the original prototype of the Wuhan-Hu isolated virus, so new variants induced uncertainty to the efficacy of the current vaccines in preventing COVID-19. To address this, we exanimated the cross-reactivity of vaccinated mouse serum against the dominant pandemic D614G variant and the five variants of concern: B.1.1.7 (Alpha), B.1.351 (Beta), P1 (Gamma), B.1.617.2 (Delta), and B.1.1.529 (Omicron). We found no significant loss of neutralizing activity against D614G, Alpha, and Gamma variants, and a lower but still neutralizing response against the Beta, Delta, and Omicron variants in ASD254-vaccinated mouse serum collected 1 year after immunization. In clinical studies, serum obtained from volunteers immunized with two doses of mRNA-1273 vaccine (Moderna) or BNT162b2 (Pfizer/BioNTech) showed equal neutralizing activity against WT, Alpha, and Gamma variants, with a 7- to 10-fold reduction against the Beta variant with mRNA-1273; a 5- to 10-fold reduction against the Beta variant and a 6-fold reduction against the Delta variant with BNT162b2 [52–56]. However, some vaccines lost neutralizing antibodies against variants, and the 50% neutralizing titers gradually decreased after 3 months with mRNA-1273 vaccination, even though the neutralizing fold change remained similar at different time points within 6 months [52]. Although serum obtained after two doses of AZD1222 vaccine (AstraZeneca) also showed a 9-fold reduction between the WT and Beta variant, about 36% (9/25) of vaccinated serum samples lost neutralizing activity, probably because of lower neutralization titers induced [55].

In this study, we also demonstrated that vaccination with ASD254 generated durable humoral immune responses without any decline in either RBD-specific antibodies or neutralizing antibodies during 1 year of monitoring. Though the mechanisms involved in causing long-term antibody responses are still not fully understood, the durability of antibody responses appears to be determined by the repetitive nature of the antigen [57]. Multivalent display, such as nanoparticle vaccines, provides more durable immunity than monovalent antigens. Moreover, we conducted a toxicity study that followed the good laboratory practice standards in Sprague Dawley rats and found that there were no clinical changes other than the injection site-related erythema and edema. There appeared to be no remarkable or irreversible adverse effect associated with ASD254. Taken together, we have demonstrated that encapsulating RBD protein into the ASD25x vaccine platform could efficiently generate a potent COVID-19 vaccine candidate that is scalable, easy to manufacture, stable, and immunogenic. The vaccinated mouse generated durable high titers of neutralizing antibodies, which also showed cross-protection activity against variants of concern and efficiently protected mice against SARS-CoV-2 challenge.

## Data Availability

All relevant data supporting the findings of this study are available within the paper and its supplementary information files.
